# Predicting Bulk Average Velocity with Rigid Vegetation in Open Channels Using Tree-Based Machine Learning: A Novel Approach Using Explainable Artificial Intelligence

**DOI:** 10.3390/s22124398

**Published:** 2022-06-10

**Authors:** D. P. P. Meddage, I. U. Ekanayake, Sumudu Herath, R. Gobirahavan, Nitin Muttil, Upaka Rathnayake

**Affiliations:** 1 Department of Civil and Engineering, University of Moratuwa, Moratuwa 10400, Sri Lanka; sumuduh@uom.lk; 2 Department of Computer Engineering, University of Peradeniya, Galaha 20400, Sri Lanka; imeshuek@eng.pdn.ac.lk; 3 Department of Civil and Environmental Engineering, University of Ruhuna, Matara 81000, Sri Lanka; gobirahavan.r@cee.ruh.ac.lk; 4 Institute for Sustainable Industries & Liveable Cities, Victoria University, P.O. Box 14428, Melbourne, VIC 8001, Australia; 5 College of Engineering and Science, Victoria University, P.O. Box 14428, Melbourne, VIC 8001, Australia; 6 Department of Civil Engineering, Sri Lanka Institute of Information Technology, Malabe 10115, Sri Lanka; upaka.r@sliit.lk

**Keywords:** bulk average velocity, explainable artificial intelligence, rigid vegetation, tree-based machine learning

## Abstract

Predicting the bulk-average velocity (U_B_) in open channels with rigid vegetation is complicated due to the non-linear nature of the parameters. Despite their higher accuracy, existing regression models fail to highlight the feature importance or causality of the respective predictions. Therefore, we propose a method to predict U_B_ and the friction factor in the surface layer (f_S_) using tree-based machine learning (ML) models (decision tree, extra tree, and XGBoost). Further, Shapley Additive exPlanation (SHAP) was used to interpret the ML predictions. The comparison emphasized that the XGBoost model is superior in predicting U_B_ (R = 0.984) and f_S_ (R = 0.92) relative to the existing regression models. SHAP revealed the underlying reasoning behind predictions, the dependence of predictions, and feature importance. Interestingly, SHAP adheres to what is generally observed in complex flow behavior, thus, improving trust in predictions.

## 1. Introduction

**Figure 1:**
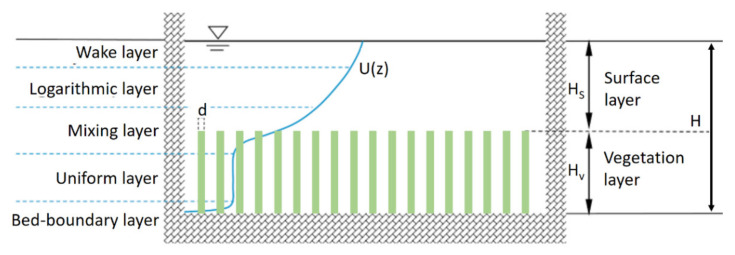
Schematic diagram of flow with vegetation; source: Shi et al. [4].

Flow-through vegetation is often observed in rivers and channels. The condition is particularly common during floods. However, the interaction between flow and vegetation provides a complicated flow field. Therefore, understanding and analyzing flow situations is highly important for various engineering and management aspects of these rivers and channels. The literature showcases many studies related to understanding the flow situation across these water bodies. Huai et al. [1], Nikora et al. [2], and Tang et al. [3] attempted to examine the velocity (vertical) distribution of an approaching flow through vegetation. They had established mathematical models to express the velocity variation. Nikora et al. [2] specified five layers that can be observed in a complex flow regime: namely, bed-boundary, uniform, mixing, logarithmic, and wake (refer to Figure 1). The first layer is the closest to the channel bed. It is generally thin, and a rapid increase in the longitudinal velocity can be observed with height above the bed. The second layer, called uniform, has a state of equilibrium from sliding forces and drag forces. Subsequently, a complicated layer (mixing) can be identified. Accordingly, the chaotic nature of flow makes the velocity distribution difficult to predict.

By employing the fundamentals of river engineering, flood discharge can be evaluated solely in terms of bulk mean velocity ($U_{B}$). However, as a result of the complicated flow field, predicting $U_{B}$ is extremely difficult under submerged vegetation conditions. These vegetations remarkably alter the hydrodynamics of flow [4]. Hence, the research community has explored the applicability of numerical expressions to predict $U_{B}$. For example, various single-layer and multi-layer approaches were introduced [4].

Cheng [5] used the Darcy–Weisbach formula to derive a single-layer model. They included provisions to consider the hydraulic radius and vegetation obstructions. In addition, a relationship was constructed between the Darcy–Weisbach coefficient, energy slope, vegetation density, and submergence. Tinoco et al. [6] employed genetic programming (GP) to search for an acceptable mathematical expression in which the Froude number was set as a target parameter. A Chezy-like formula was found as the final expression. In addition, Gualtieri et al. [7] examined distinct conventional equations in flow with vegetation and high submergence. They reported that Keegan’s equation is one of the best-performing models. Generally, these single-layer models are simple in that the effects of vegetation-induced drag and roughness-induced resistance are ignored. Furthermore, the flow resistance equations developed by Cheng [5], Gualtieri et al. [7], and Tinoco et al. [6] were established in flumes, neglecting vegetation. However, friction resistance was considered, which occurs as a result of bed roughness in those equations. Nevertheless, in the flow-through vegetation, drag is a dominant source of resistance in contrast to bed roughness [8]. Bed-induced roughness is markedly different from vegetation-induced roughness. Therefore, the direct application of traditional equations is due attention. 

In contrast, two-layer models separate flow from vegetation into the resistance layer, which considers the vegetation layer. The top of the vegetation layer is the lower boundary of the surface layer. Subsequently, the velocity in each layer ($U_{V}\;\left(\mathrm{vegetation}\;\mathrm{layer}\right)$ and $U_{S}\;\left(\mathrm{surface}\;\mathrm{layer}\right)$) is estimated, and $U_{B}$ is derived using a combination of weights. The average velocity in the vegetation layer is usually calculated using force balance between sliding force and vegetation-induced drag force [8,9,10,11,12]. In addition, velocity in the surface layer is estimated using a logarithmic assumption [12] based on equations similar to the Darcy–Weisbach [10] or equivalent considerations [8]. In addition, Kolmogorov’s theory of turbulence [13], genetic programming [9], and representative of roughness height [5,10] were also used in the literature. 

As these expressions highlight, two major parameters that affect flow resistance are submergence and non-dimensional vegetation density. Huthoff et al. [8], Augustijn et al. [14], Nepf [15], and Pasquino et al. [16] reported that the shear layer was similar as a result of the increase in the submergence ratio ($H/H_{V}>5$; H—Total flow height and $H_{v}—\mathrm{Height}\;\mathrm{of}\;\mathrm{vegetation}$). Belcher et al. [17] have described three distinct flow regimes based on the density of vegetation (λ << 0.1, λ = 0.1, and λ > 0.23, where λ is the density of vegetation). For λ << 0.1 (sparse vegetation), the shear layer again resembles a boundary layer, whereas a shear layer resembling a free layer with an inflection point can be observed for transitional (λ = 0.1) and dense (λ > 0.23) vegetation. Recently, Shi et al. [4] combined the two-layer approach and GP to develop analytical models to predict U_B_. In addition, they proposed an equation to predict the friction coefficient (f_S_) for the surface layer. 

However, these equations and methods failed to achieve a mean relative error (MRE) within 10% [4]. Be that as it may, developing such integrated formulae is less practical. Numerical modeling generally requires significant time and effort. Moreover, previous equations demonstrate that the relationship is extremely non-linear. According to GP-based studies, in order to explore such complex relationships, ML can be applied as it is fast and requires less effort. Unique model architectures are available that can approximate complex relationships. Many researchers have used ML in the hydrology field [18,19,20,21]. Generally, ordinary and ensemble learning methods have been frequently used to examine complex relationships. However, it was reported that ensemble models are highly efficient in hydrological modeling [22,23,24].

For example, Cannon and Whitefield [25] examined changes in streamflow as a result of climate change using multiple linear regression (MLR) and ensemble neural networks (ENN). Accordingly, the ENN model performed better in contrast to the MLR method (4% improvement in R^2^). Diks and Vrugt [26] used model averaging ensemble methods to forecast streamflow in the Leaf River watershed, Mississippi. They proposed Granger–Ramanathan averaging (GRA) as the superior model averaging method in their study. Later, Li et al. [27] reported that ensemble methods consisting of bagged-MLR and bagged-support vector machines (SVM) outperformed individual ML models. Tiwari and Chatterjee [28] attempted to predict daily river discharge using bootstrap and wavelet artificial neural networks (ANN). Similarly, the combined model showcased its superiority in predicting streamflow with respect to the individual models. Erdal and Karakurt [29] employed tree-based learners (classification and regression trees (CART)) to build ensemble models (bagged regression tree (BRT), stochastic gradient-boosting regression trees (GBRT)). The study showed that BRT and GBRT are better than the CART and SVM models (17% improvement in RMSE indices). Kim et al. [30] proposed a method to estimate discharge using satellite altimetry data using ensemble regression ML. They combined conventional rating curves with the ensemble method, and it was effectively used to predict discharge in the Congo River. 

Alternatively, ML is popular in predicting extreme events such as droughts [31,32] and flood events. For instance, several studies focused on predicting floods and geospatial mapping of flood susceptibility with the aid of ML [33,34,35]. Shu and Burn [36] evaluated the flood index using ANNs. They concluded that ensemble ANNs were significantly (10% improvement in relative squared error) reliable compared to individual ANNs. Araghinejad et al. [37] reported that a 50% improvement in precision was obtained for ensemble ANNs in predicting floods. Lee et al. [38] developed boosted tree models and random forests for flood mapping. The random forest model provided moderately better results compared to boosted tree models. Recently, Arabameri et al. [33] claimed that ensemble methods provide excellent accuracy in flash flood susceptibility mapping. 

Unlike surface hydrology, hydrogeology has to deal with a shortage of data. These processes show a highly non-linear nature; therefore, accurate predictions are greatly important. Singh et al. [39] developed tree-based models to reproduce groundwater hydrochemistry in the north Indian region. Repeatedly, ensemble models were effective in contrast to single tree models. Ref. [40] evaluated the performance of wavelet-based ML models (extreme learning machine (ELM), the group method of data handling (GMDH), and wavelet ANN) to predict groundwater levels. Similarly, several attempts stated the superior performance of ensemble models in hydrogeology [41,42]. 

However, none of these studies highlighted the human comprehensibility of ML predictions. For example, regardless of higher accuracies, predictions are unexplainable and contain an explicit black box. Despite hyperparameters tuned during model training, the end-user is not aware of the inner-working methodology of the ML model. Further, the end-user does not know which parameters are significant for a particular prediction. Such drawbacks weaken the user’s confidence in ML-based predictions. Moreover, it prevents the implementation of ML models in real-life applications in hydrology. 

Explainable artificial intelligence (XAI) intends to eliminate the previously mentioned drawbacks of ML. XAI helps to identify important parameters, revealing the inner-working principle of the ML model. Therefore, it provides a better understanding to the end-user of decision making. XAI is becoming popular in many fields (e.g., data science, business, engineering) as a result of the human comprehensibility explanation [43]. Accordingly, XAI turns a *black box* model into a *glass box* model, thereby elucidating the working principle and causality behind predictions [44,45]. A few studies used explainable/interpretable ML to predict evapotranspiration and estuarine water quality [46,47].

To the best of the authors’ knowledges, no related studies have been conducted to predict bulk average flow (with vegetation) using explainable ML. The objective of this study is to investigate the performance of the interpretable ML in order to improve the bulk average flow predictions in contrast to conventional regression models. Moreover, as a core part of the study, the same approach is used to predict the friction coefficient of the surface layer (f_S_). Therefore, the present study is imperative and novel as it: (a) engages tree-based ML models in the prediction of U_B_ and f_S_, and (b) interprets the inner-workings of ML models to improve the end-users’ confidence. The interpretable ML distinguishes influencing parameters and provides instances and global explanations of the model. On one hand, this is significant because it cross-validates ML predictions using experimental data. Overall, the study emphasizes that XAI does not essentially require sacrificing precision or complexity but rather supports the model’s predictions by providing human-understandable interpretations. 

Since the hydrology research community is new to XAI, the authors start by introducing XAI and subsequently, in Section 2, describe the specific interpretation model we used: SHAP (Shapley Additive exPlainations). Section 3 describes the ML models that we employed for the study. Section 4 provides data description and working methodology, and Section 5 consists of a performance evaluation of ML models. The novel ML interpretations are provided in Section 6. Section 7 concludes the paper, and the limitations and future work of this study are presented in Section 8.

## 2. Explainable Artificial Intelligence (XAI)

**Figure 2:**
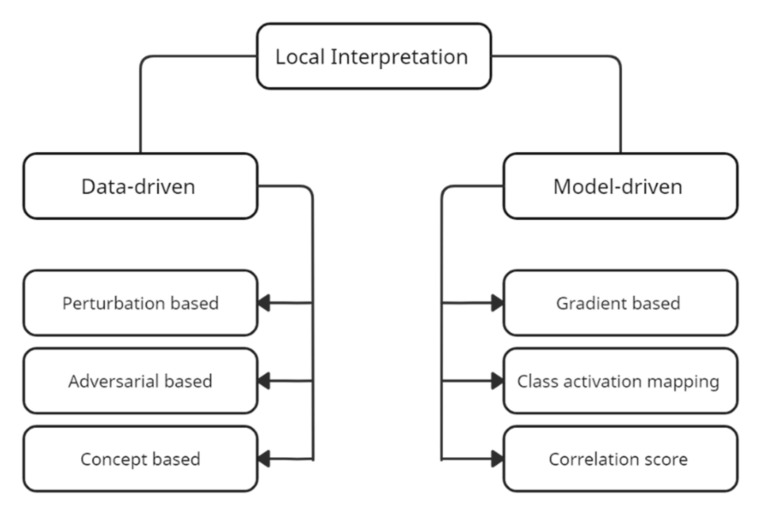
Classification of ML interpretation methods.

As previously highlighted, ML-based predictions require transparency to advance the end-users’ confidence [48,49]. According to Lundberg and Lee [50], the best explanation for a model is the model itself. For example, models such as decision trees with lower tree depths are self-explanatory. As the tree grows into deep layers, the model and the explanation become moderately complex. For complex models whose inner workings are explicitly unknown, post-hoc explanations are recommended. Such explanations strongly contribute to advancing the decision-making and providing underlying reasonings behind predictions. Figure 2 shows the summarized classification of explanation methods.

Data-driven and model-driven explanations are the main categories of local interpretation methods [51]. For example, model-driven methods investigate the inner components of ML models, which do not need a global interpretation of the inner-working methodology of the model. The explanation depends on a category to interpret how the model performs a provided task. These models are computationally inexpensive and convenient to implement. Moreover, these models are subdivided as such into a class activation map [52,53,54], gradient-based interpretation [55,56], and correlation-score interpretation [57,58,59,60].

Data-driven explanations depend on inputs for the interpretation process; however, this does not necessitate understanding the working principle inside the ML model. It scrutinizes the effects of deviations in each input data on the ML model. There are three sub-sections under data-driven interpretations, namely, concept-based interpretations [61,62], adversarial-based interpretations [63,64], and perturbation-based interpretations [56,65,66]. The authors suggest perturbation-based interpretations for the present study.

Perturbation works by masking a segment of the input data of the ML model. Masking separate regions provides a set of disturbances. Afterward, the disturbed set is taken to the model to obtain a new set of predictions. Later, the original predictions are compared with the predictions obtained using a disturbed sample. Accordingly, the significance of input data (different segments) is obtained. Within each perturbation method, unique strategies and explanation rules are observed. For example, perturbation methods consist of several models such as CXplain, LIME [67], RISE [68], and SHAP [50]. The authors notice that SHAP and LIME are frequently used in ML-based studies. These two methods differ from the method used to calculate weights. LIME creates dummy instances and weighs the instances based on their similarity. SHAP used Shapley values to estimate the weight of each sampling instance. As a result of dummy instances, Moradi and Samwald [69] argued that LIME does not provide the actual feature value, but rather considers the neighborhood of a particular instance. SHAP provides a unified measure of feature importance compared to LIME. Therefore, we recommend SHAP explanations to elucidate tree-based models and their predictions.

### SHAP (Shapley Additive Explanations)

Lundberg and Lee [50] suggested using SHAP to elucidate ML predictions based on game theory. For example, inputs are referred to as players, while prediction becomes payout. SHAP determines the contribution of each player to the game. Lundberg and Lee [50] have introduced several versions of SHAP (e.g., DeepSHAP, Kernel SHAP, LinearSHAP, and TreeSHAP) for specific ML model categories. For example, TreeSHAP is used in the present study to explain ML predictions. It uses a linear explanatory model and Shapley values (Equation (1)) to estimate the initial prediction model.

(1) $$f\left(y^{\prime }\right)={ɸ}_{o}+\sum_{i=1}^{N}{ɸ}_{i}{y^{\prime }}_{i}$$

where f denotes the explanation model, and $y^{\prime }\in {\left\{0,1\right\}}^{N}$ denotes the simplified features of the coalition vector. N and ɸ∈ℝ denote the maximum size of the coalition and the feature attribution, respectively. Lundberg and Lee [50] provided Equations (2) and (3) to calculate the feature attribution.

(2) $${ɸ}_{i}=\underset{S\subseteq \left\{1,\ldots ,p\right\}\backslash \left\{i\right\}}{\sum }\frac{\left|S\right|!\left(p-\left|S\right|-1\right)!}{p!}\left[g_{x}\left(S\cup \left\{i\right\}\right)-g_{x}\left(S\right)\right]$$

(3) $$\mathrm{where};\;g_{x}\left(S\right)=E\left[g\left(x\right)│x_{S}\right]$$

In Equation (2), S represents a subset of the features (input), and x is the vector of feature values of the instance to be interpreted. Thus, the Shapley value is denoted through a value function $(g_{x}$). Here, p symbolizes the number of features; $g_{x}\left(S\right)$ is the prediction obtained from features in S; and $E[g\left(x\right)│x_{K}]$ represents the expected value of the function on subset S. 

## 3. Data Description

The data set was obtained from a series of studies conducted by [70,71,72,73,74,75,76,77]. Even though 315 instances were available, 27 instances are theoretically inexplainable (U_v_ (velocity in vegetation layer) > U_s_ (velocity in the surface layer)). Therefore, the remaining 288 instances were employed for ML [4]. Descriptive statistics are summarized in Table 1. 

The data set is simultaneously used to: (a) predict f_S_ (friction coefficient in the surface layer) and (b) predict U_B_ (bulk average velocity). From the experimental data, the following Equation (4) was used to calculate U_B_. All parameters are presented in Table 1. Equation (5) was used to calculate f_S_.

(4) $$U_{B}=\frac{Q}{\left(B\left(1-\lambda \right)H_{v}+H_{s}\right)}$$

where Q is the measured flow(m^3^/s), H_S_ is theheight of surface layer (m), H_V_ is the height of vegetation layer (m), U_B_ is the bulk average velocity (m/s), B is the channel width (m), and λ is the vegetation density.

**Figure 3:**
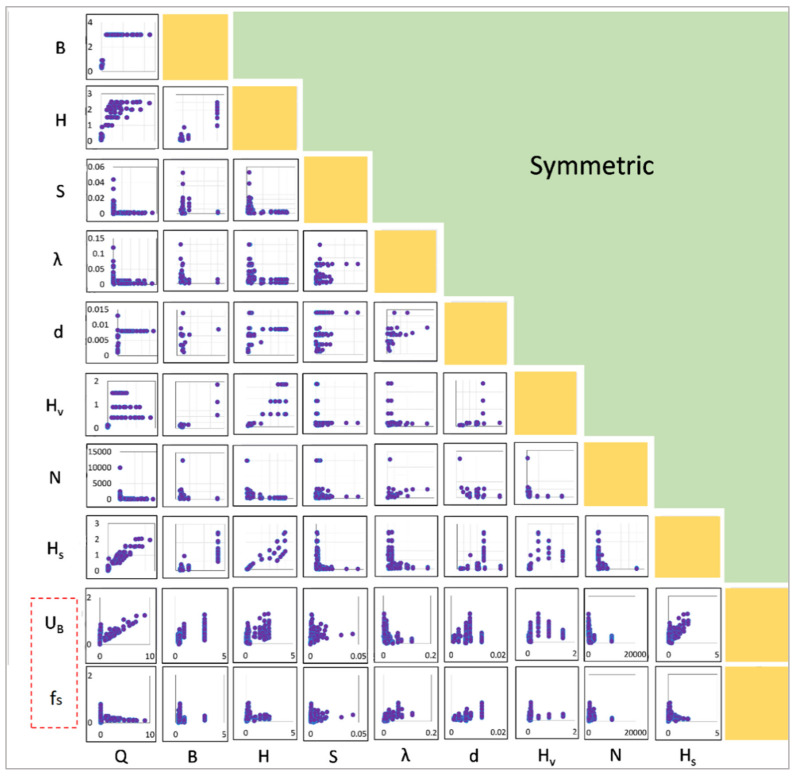
Pairwise correlation plot. (All of the variables’ (dependent and independent) parameters are plotted against each other. Labels are located at the bottom of the figure, and the scale denotes the x-axis for all boxes along their respective column. Labels are on the left, and the scale denotes the y-axis for all boxes along their respective row.

Figure 3 shows the pairwise plot of each variable. Accordingly, Q and H_s_ have a moderate correlation with U_B_. The remaining parameters show an explicitly non-linear behavior with U_B_. Further, none of the parameters show a good correlation with f_S_. Therefore, simple models, such as linear regression, are inadequate for building a relationship. The present study employed tree-based models to predict U_B_ and f_S_.

However, previously introduced equations (to predict U_B_) do not consist of Q and more often ‘d’. In addition, the term H can be linearly expressed as H_s_ + H_V_. Therefore, we neglect parameters Q, d, and H for the ML model. U_B_ is supposed to be a function of S, λ, B, H_V_, N, and H_S_. Equations (8)–(14) are several existing regression models developed to predict U_B_. Shi et al. [4] assumed that f_S_ = g(S, λ, H/H_V_, and aH_V_), where a = 4λ/πd. However, the solution they obtained only consists of λ (refer to Equation (15)). 

Given previous assumptions, we suggest the relationships f_S_ = f (S, λ, d, H_V_, N, and H_S_) and U_B_ = f (S, λ, B, H_V_, N, and H_S_) for machine learning models.

(5) $$f_{s}=\frac{8{\mathrm{gH}}_{S}S}{U_{s}{}^{2}}$$

where f_S_ is the friction coefficient in the surface layer, g is the gravitational acceleration (ms^−2^), S is the energy slope, and U_S_ is the velocity in the surface layer (m/s).

(6) $$U_{s}=\frac{Q-U_{V}\left(1-\lambda \right)H_{v}B}{{\mathrm{BH}}_{s}}.$$

U_V_—Velocity in vegetation layer (m/s).

(7) $$U_{v}=\sqrt{\frac{2\mathrm{gS}\left(1-\lambda \right)+\frac{H_{s}}{H_{v}}}{C_{d\left(\mathrm{Cheng}\right)}a}}$$

a = 4λ/πd, C_d_ = drag coefficient.

Cheng [5]

(8) $$U_{B}=2.1R_{*}^{0.1}{\left(\frac{H}{H_{v}}\right)}^{0.75}\sqrt{{\mathrm{gR}}_{\left(\mathrm{Cheng}\right)}S}$$

H-Flow depth (m).

(9) $$R_{*}=R_{\left(\mathrm{Cheng}\right)}{\left(\frac{\mathrm{gS}}{{ʋ}^{2}}\right)}^{\frac{1}{3}}$$

(10) $$R_{\left(\mathrm{Cheng}\right)}=\frac{{\mathrm{BH}}_{v}\left(1-\lambda \right)+\mathrm{BH}}{2H+B\left(1-\lambda \right)+{\mathrm{NB}\pi \mathrm{dH}}_{v}}$$

ʋ is the kinematic viscosity of the fluid. R refers to the hydraulic radius.

Huthoff et al. [8]

(11) $$U_{B}=\left[\frac{H_{s}}{H}{\left(\frac{H_{s}}{\left(\sqrt{\frac{\pi }{4\lambda }}-1\right)d}\right)}^{\frac{2}{3}\left[1-{\left(\frac{H_{v}}{H}\right)}^{5}\right]}+\sqrt{\frac{H_{v}}{H}}\right]\sqrt{\frac{\pi \mathrm{gdS}}{2C_{d}\lambda }}$$

$C_{d}$ is the drag coefficient, which is approximately 1.0.

Shi et al. [4]

(12) $$U_{B}=\left\{\sqrt{\frac{2{\left(1-\lambda \right)}^{2}\frac{H_{v}}{H}}{C_{d\left(\mathrm{Cheng}\right)}a\left(\left(1-\lambda \right)H_{v}+H_{s}\right)}}+\sqrt{\frac{8H_{s}^{2}\frac{H_{s}}{H}}{\left(0.102+3.73\lambda \right){\left(\left(1-\lambda \right)H_{v}+H_{s}\right)}^{2}}}\right\}\sqrt{\mathrm{gHS}}$$

(13) $$C_{d\left(\mathrm{Cheng}\right)}=\frac{130}{r_{v*}^{0.85}}+0.8\left(1-e^{\frac{-r_{v*}}{400}}\right)$$

(14) $$r_{v*}={\left(\frac{\mathrm{gS}}{{ʋ}^{2}}\right)}^{\frac{1}{3}}r_{v}$$

where $r_{v}$ represents vegetation related to the hydraulic radius, and $r_{v*}$ is the non-dimensional vegetation related to the hydraulic radius.

(15) $$f_{S}=0.102+3.73\lambda$$

## 4. Machine Learning Models

The authors proposed a single ordinary method (decision tree) and two ensemble methods (extra tree and XGBoost) for this study. As highlighted in the introduction, ensemble methods result in higher efficiency than individual models. However, we intend to explain the models’ results. For the ordinary models, we used intrinsic model explanations, whereas post-hoc explanations will be used for the ensemble methods. Moreover, the two ensemble methods used here are based on the decision tree. 

### 4.1. Decision Tree Regressor

The decision tree can be introduced as the primary structure of tree-based ML algorithms. It serves either classification or regression applications. The working methodology of the decision tree is convenient to understand and interpret because it splits a complicated task into multiple simpler forms [78]. A regular decision tree structure is formed based on hierarchically arranged conditions from roots to leaves [79]. Ahmad et al. [80] provided an interesting conclusion regarding decision tree structure: it is transparent and, subsequently, can be used to generate new data sets through continual splitting. The training sequence of a decision tree model is based on recursive breakdown and multiple regression. This is initiated from the root node and continuously performed until the terminating criteria are met [81]. Each leaf node of an evolved structure can be theoretically approximated to a simple linear regression. Afterward, pruning is performed to reduce the complexity of the model and to improve the generalization.

The regression tree model attempts to distinguish data groups with the same predicted variable by examining variables. Similar to classification, the decision is made about which variables should be partitioned, the corresponding values of the partitioned variables, the number of partitions, and the decisions at terminals. Generally, the sum of the square error (SSE) is used to produce recursive splits (refer to Equation (16)). For example, per each partition, the response variable y is separated into two groups of data, R_1_ and R_2_. Subsequently, the tree operates to examine a predictor variable x with respect to the split threshold.

(16) $$\mathrm{SSE}=\underset{i\in R_{1}}{\sum }{\left(y_{i}-\bar{y_{1}}\right)}^{2}+\underset{i\in R_{2}}{\sum }{\left(y_{i}-\bar{y_{2}}\right)}^{2}$$

where $\bar{y_{1}}$ and $\bar{y_{2}}$ are the mean values of the response variables of each group. The sequence is formed for a predictor variable to minimize the SSE for the split. Thus, the tree grows with recursive splits and split thresholds similar to classification. The terminal node denotes the mean of the y values of samples collected within a node. However, there can be instances where a complex function defines a terminal node.

### 4.2. Extra Tree Regressor

The extra tree is an ensemble tree-based approach that can be used for either classification or regression [49,82]. The algorithm creates an ensemble of unpruned trees following the top-down process. The extra tree method appears different from other tree ensembles because it splits notes using random cut points. Further, it uses a whole sample to grow the tree. As a result of the random cut points and ensemble averaging, the extra tree approach can reduce variance compared to the weak randomization of other methods. In terms of computation, the complexity of the growing process is in the order of N log N concerning the learning sample size (N). Geurts et al. [83] mentioned that extra trees are approximately 1.5–3 times larger than random forests in terms of leaves. However, the complexity of the extra tree is relatively smaller since leaves grow exponentially. Moreover, the extra tree is typically faster than tree-bagging and random forests when computation time is considered.

The geometric assessment showed that the extra tree asymptotically creates piecewise continuous multi-linear functions. Therefore, the resulting models are smooth in contrast to other ensemble methods that optimize cut points. In essence, it leads to an improvement in accuracy in the regions of input space in which the target function is smooth. Geurts et al. [83] reported that the extra tree is less likely to overfit.

For regression, the extra tree uses relative variance reduction. For example, if K_i_ and K_j_ represent two subsets of cases from K corresponding to the outcome of a split, then the score can be expressed as follows (Equation (17)).

(17) $${\mathrm{Score}}_{R}\left(s,K\right)=\frac{\mathrm{var}\left\{z|K\right\}-\frac{\left|K_{i}\right|}{\left|K\right|}\mathrm{var}\left\{{z|K}_{i}\right\}-\frac{\left|K_{j}\right|}{\left|K\right|}\mathrm{var}\left\{{z|K}_{j}\right\}}{\mathrm{var}\left\{z|K\right\}}$$

$\mathrm{var}\left\{z|K\right\}$ denotes variance of outcome z in sample K.

### 4.3. Extreme Gradient Boosting Regressor (XGBoost)

XGBoost is an implementation of gradient boosting decision trees [84,85]. As the base learner, a decision tree is used for integration. Conversely, it is an ensemble algorithm formed on gradient descent iterations. Continous splits are performed to grow the tree structures. For instance, each tree (decision) computes the feature and corresponding threshold along with the best branch effect. Ultimately, predictions become more consistent. XGBoost is preferred for either classification or regression problems, and it is popular among data scientists as a result of its superior execution speed. The workflow of XGBoost is as follows.

For example, let a data set R with k features and m number of examples complete the equation: $R=\left\{\left(x_{i},y_{i}\right):i=1,2,\ldots m,x_{i}\in {\mathbb{R}}^{k},{\;y}_{i}\in \mathbb{R}\right\}$. Accordingly, we suppose $\hat{y_{i}}$ is a prediction of a model generated from the following sequence.

(18) $$A_{i}=\varphi \left(x_{i}\right)=\sum_{j=1}^{J}g_{j}\left(x_{i}\right)$$

where notation J denotes the number of trees, and $g_{j}$ represent the jth tree. To solve Equation (18), suitable functions should be found, minimizing the regularization objective ($\zeta \left(\varphi \right))$ and loss. In Equation (19), notation L represents loss function, which is the difference between the actual ($y_{i})$ and predicted outputs ($\hat{y_{i}})$. The second term measures the complexity of the model and avoids possible chances of overfitting. The extended version of the complexity term $\left(Ω(g_{j}\right))$ is expressed in the following Equation (20).

(19) $$\zeta \left(\varphi \right)=\underset{i}{\sum }L(y_{i},A_{i})+\underset{j}{\sum }Ω(g_{j})$$

(20) $$Ω(g_{j})=\Upsilon T+0.5\vartheta ||w|{|}^{2}$$

where T, in Equation (20), denotes the number of leaf nodes, and w is the weight of a leaf. Boosting is used for the training model to minimize objective function, and a new function is added during model training. Here, Υ denotes the difficulty in node segmentation, and ϑ is the L2 regularization coefficient. Since XGBoost is also a decision tree-based model, multiple hyper-parameters, including sub-sample and maximum depth, are employed to reduce overfitting, further enhancing the performance. 

## 5. Performance Evaluation of Tree-Based Models

**Figure 4:**
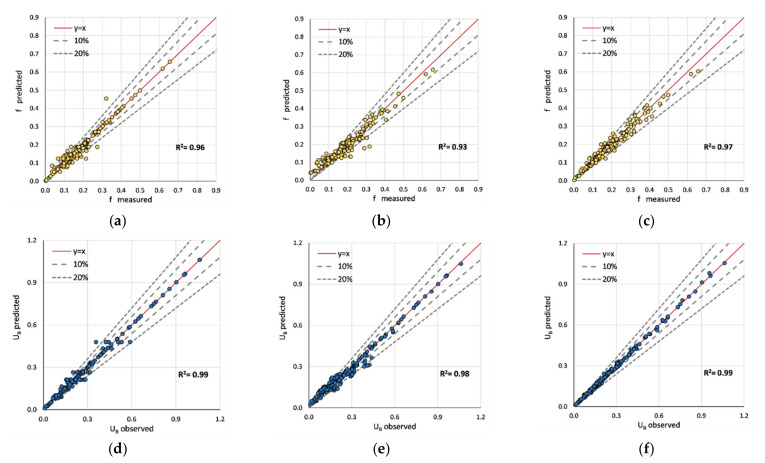
Comparison of training accuracies of ML models (both f_S_ and U_B_): (**a**) decision tree regressor; (**b**) extra tree regressor; (**c**) XGBoost regressor; (**d**) decision tree regressor; (**e**) extra tree regressor; (**f**) XGBoost regressor.

For model training, 70% (201 out of 288 instances) of the sample was employed while the remaining was used for validation. R^2^ was scrutinized as the training score. Figure 4 depicts the summary of the training process for each model. All models are accurate and deviate less from the observed values. However, the training process of f_S_ holds less consistency compared to the process of U_B_. In addition, the highest training score (R^2^) was obtained from the XGBoost model for f_S_ and U_B_. Even so, the weakest decision tree obtains a 0.99 training score for U_B_. Accordingly, ML models have learned the non-linearity associated with each sequence, separately. Hyper-parameters were simultaneously optimized using a *grid search*. Grid search methods create numerous models by using different combinations of hyperparameters. Eventually, the models will be evaluated to obtain the optimum hyperparameters based on prediction accuracies. The effect of each hyperparameter is separately illustrated in Appendix A.

**Figure 5:**
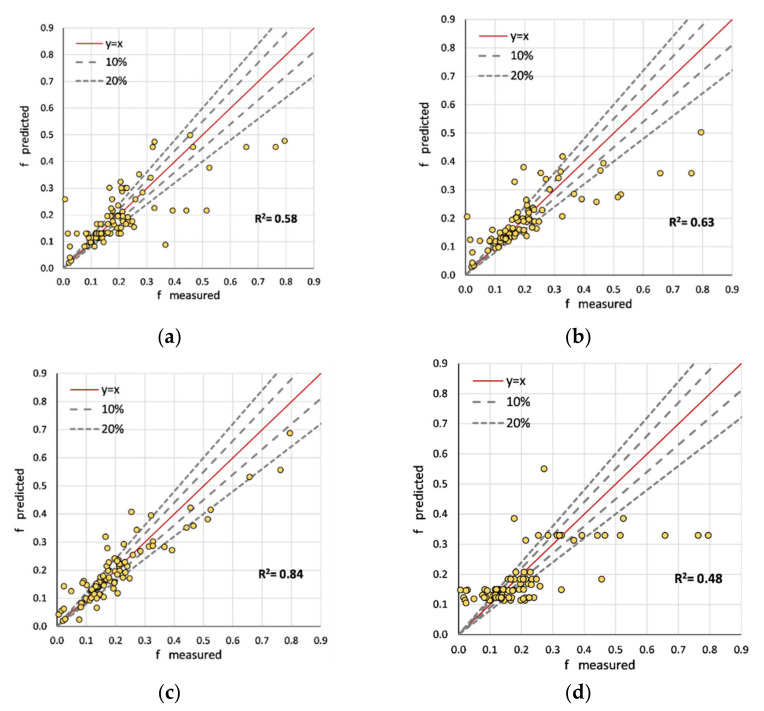
Comparison of validation accuracies of predicted f_S_: (**a**) decision tree regressor; (**b**) extra tree regressor; (**c**) XGBoost regressor; (**d**) Shi et al. [4].

In order to apply XAI, the validation accuracies of the predictions (87 instances) of tree-based models were estimated by comparing the f_S_ and U_B_ values. In addition, the equation proposed by previous authors was used for comparison. Figure 5 shows the comparison of friction coefficient (f_S_) predictions. Both the extra tree and decision tree regressor obtained moderately lower accuracies compared to the training sequence. The authors emphasize that a large deviation between training and validation occurs as a result of overfitting. Using a large number of data samples will eliminate the issues due to overfitting. Accordingly, we suggest the XGBoost model as the superior model (R^2^ = 0.84). The model introduced by Shi et al. [4] failed to surpass the accuracy of the weakest tree model. Despite the training sequence, the validation shows a distinct attribute of each model. For example, the equation suggested by Shi et al. [4] has more deviations for predictions closer to zero compared to the tree-based models. Both the extra tree and decision tree have predictions that exceed the 20% error margin. However, the XGBoost model, at a considerably lower tree-depth, more often provides predictions, thereby avoiding such inconsistencies.

**Figure 6:**
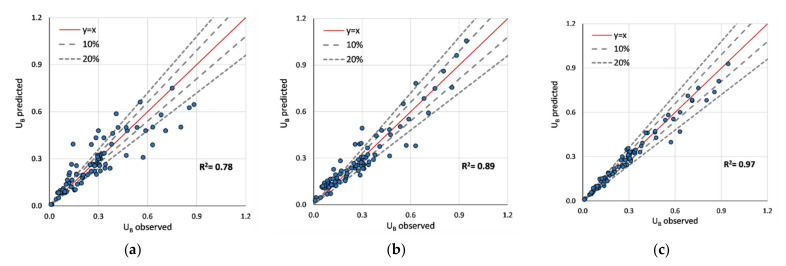

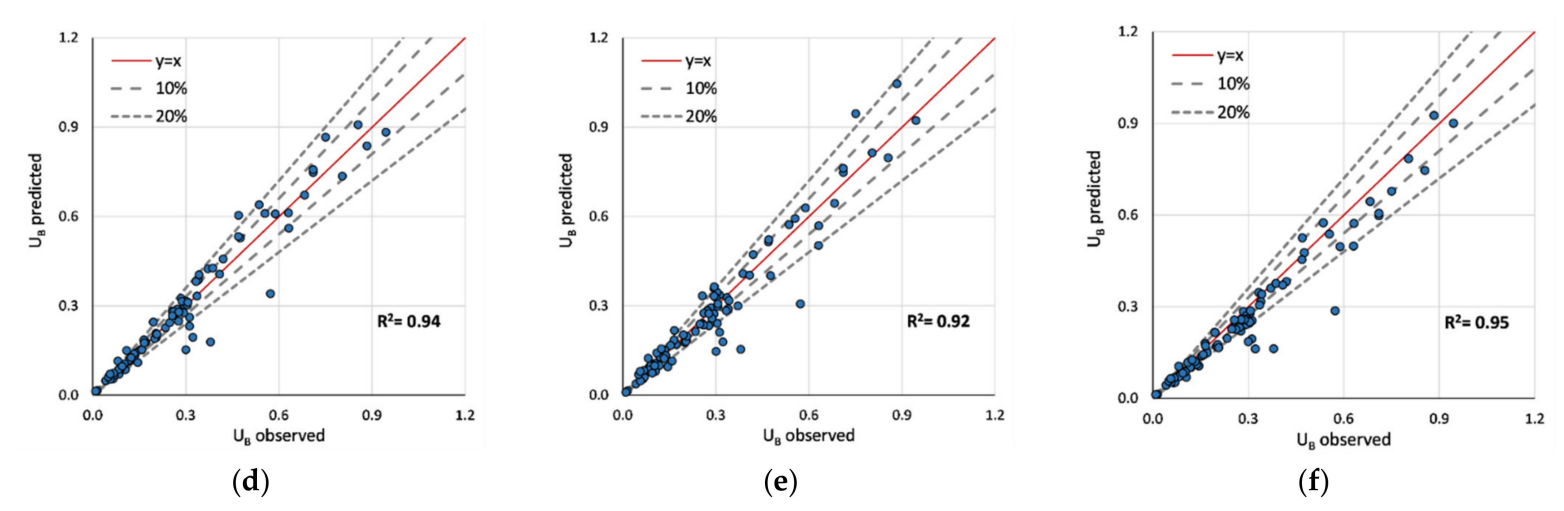
Comparison of validation accuracies of predicted U_B_: (**a**) decision tree regressor; (**b**) extra tree regressor; (**c**) XGBoost regressor; (**d**) Shi et al. [4]; (**e**) Cheng [5]; (**f**) Huthoff et al. [8].

Figure 6 depicts the validation predictions (U_B_) obtained from different models. The first row consists of the ML predictions, whereas the latter consists of those of the existing regression models. Among the models, the highest accuracy was obtained for the XGBoost (R^2^ = 0.97) model, and the lowest was obtained for the decision tree model (R^2^ = 0.78). Given the conclusions of Shi et al. [4], the model proposed by Huthoff et al. [8] fits better with the validation set. The equation from Cheng [5] achieved an R^2^ of 0.92. Compared to Huthoff’s model, XGboost shows fewer deviations in predictions. For example, 0.3 < U_B_ < 0.6 regions consist of inconsistencies as shown in Figure 6f. However, the observed inconsistencies are inferior to the XGBoost model. The two equations proposed by Shi et al. [4] and Cheng [5] provide conservative values compared to Huthoff’s equation.

All prediction accuracies were numerically evaluated using the equations provided in Appendix B (refer to Table 2). The obtained R values show how well predictions fit into the experimental observations. All ML predictions exceeded R = 0.8 for U_B_ and R = 0.75 for f_S_, indicating a strong correlation between predictions and observations. Subsequently, the R^2^ value indicates different degrees of the deviations of ML predictions in contrast to the actual values. For example, the decision tree had more deviations from the experimental data than the rest of the ML models. However, the XGBoost model showed superior performance to all of the models, including previous regression models. Shi’s and Huthoff’s equations reached a good R^2^ value. Still, the R^2^ value of XGBoost is higher than the the R^2^ value of those two models. This explains the better flexibility of the XGBoost algorithm to perform a task similar to those complex regression models. The MAE and RMSE values obtained for XGBoost and Huthoff’s equation are 0.025, 0.04, and 0.038, 0.06, respectively. The authors suggest fractional bias values between −0.3 to 0.3 for an acceptable model. Accordingly, the negative fractional bias value indicates that ML models underestimate predictions that cause slight imperfections. 

**Figure 7:**
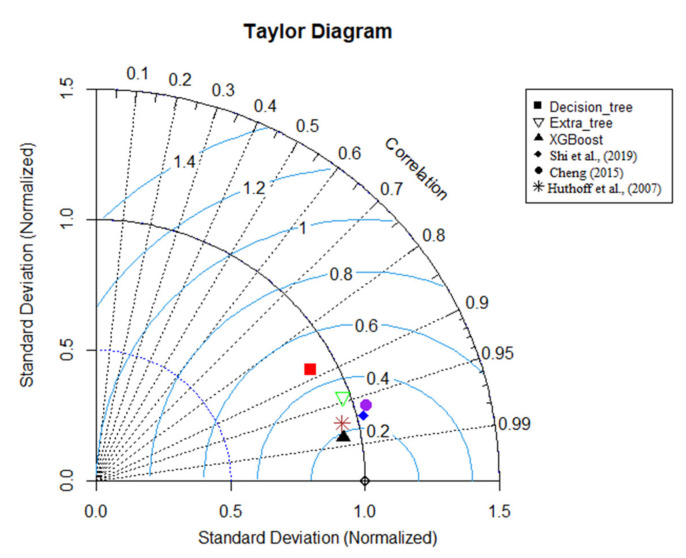
Taylor diagram for U_B_ predictions; Huthoff et al. [8], Shi et al [4], and Cheng [5].

**Figure 8:**
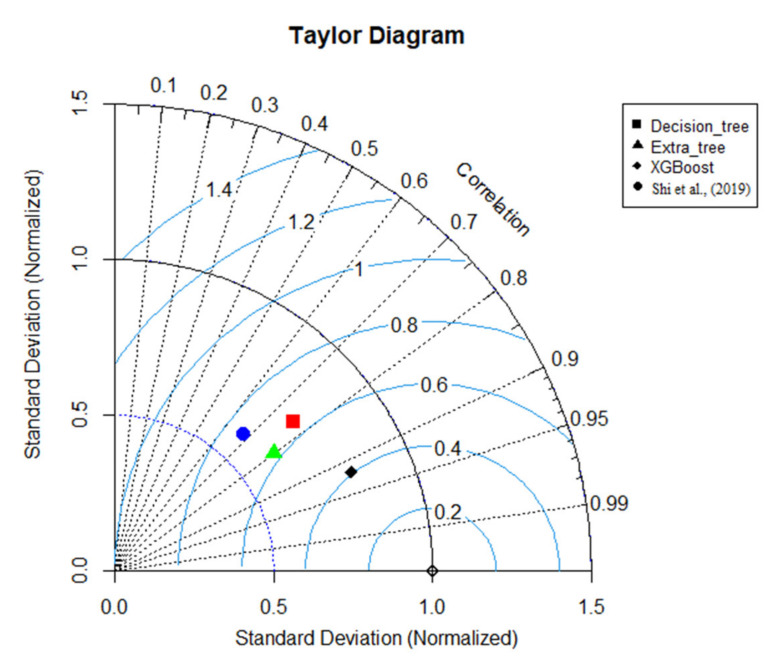
Taylor diagram for f_S_ predictions; Shi et al [4].

However, the extra tree and decision tree were not perfect for predicting f_S_. Still, those three models are superior in contrast to the equation proposed by Shi et al. [4]. For instance, Shi’s equation obtained the highest MAE and RMSE values (0.069 and 0.11). Compared to U_B_ predictions, the f_S_ predictions achieved moderately less accuracy, despite the XGBoost predictions. XGBoost obtained an R^2^ value of 0.85 and an MAE value of 0.042 for validation predictions. Moreover, it captured variation better than the existing equation. Figure 7 and Figure 8 show Taylor diagrams of validation predictions for both occasions. Accordingly, the XGBoost model is superior to the other models in both tasks with a strong correlation. However, for U_B_ predictions, Huthoff’s model is also comparable, but slightly less accurate, in contrast to the XGBoost model.

**Figure 9:**
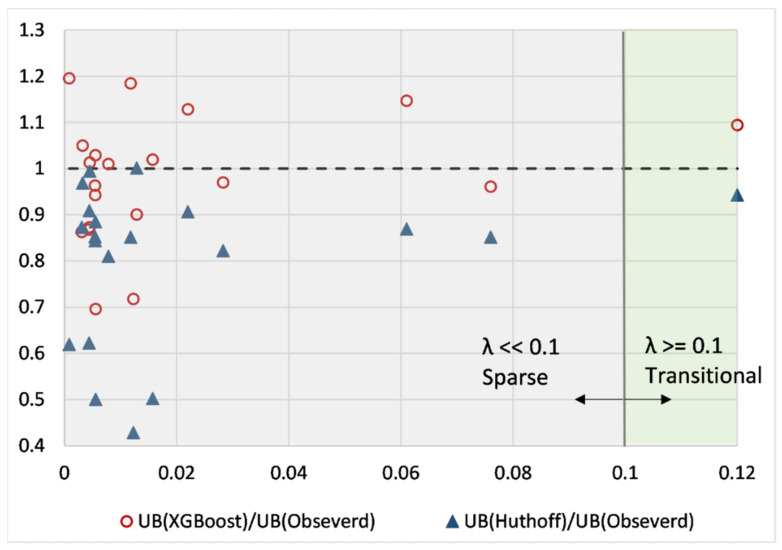
Comparison of XGBoost and Huthoff’s model in different flow regimes.

In addition, Belcher et al., [17] and Nepf [15] introduced three flow regimes based on λ. According to the current data set, λ << 0.1 will be considered sparse vegetation, whereas λ ≥ 0.1 is considered transitional (note λ > 0.23 dense). We intend to compare the XGBoost predictions and Huthoff’s predictions according to these flow regimes (refer to Figure 9). Since the analysis of this study emphasized that Huthoff’s model is superior to existing regression models, it is observed that XGBoost consists of a higher prediction accuracy (with lower deviations) in sparse vegetation conditions. In the same regime, deviations of up to 60% can be expected from Huthoff’s regression model. However, from sparse vegetation to transition regime, both models show a comparable prediction accuracy for U_B_.

**Figure 10:**
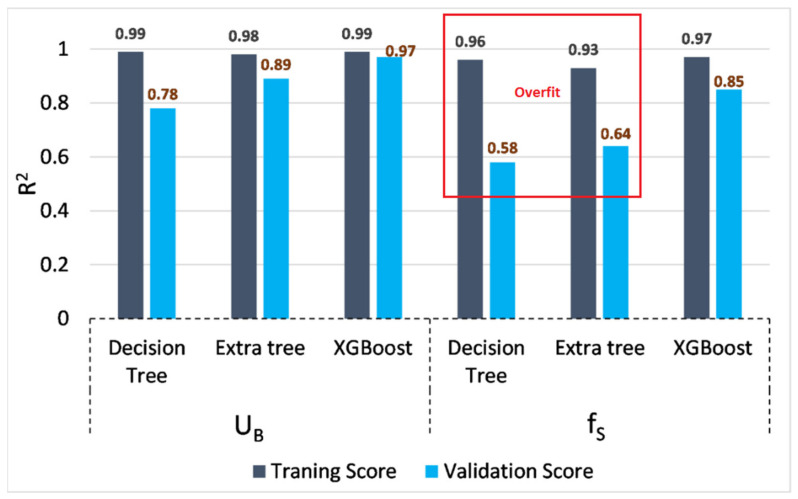
Training and validation accuracy of ML models (optimized using hyperparameters).

As previously mentioned, ML models can overfit a training data set, which can result in higher and lower prediction scores for training and validation sequences, respectively. Therefore, the authors examine this drawback using the R^2^ score of training and validations (refer to Figure 10). Accordingly, all three models are acceptable for predicting U_B_ with rigid vegetation. However, the decision tree’s and extra tree’s performances are suspicious for predicting f_S_. For example, the gap between validation and training scores is significant for both these models. Therefore, considering abrupt changes in the decision tree and extra tree when predicting f_S_, the authors do not recommend these two models to obtain further validation predictions. However, the validation scores were better than the observed score for Shi’s equation. On one hand, it shows the unique prediction performance of each tree-based model, despite them being decision tree-based algorithms.

## 6. Application of XAI for Model Predictions

### 6.1. Intrinsic Model Explanation

**Figure 11:**
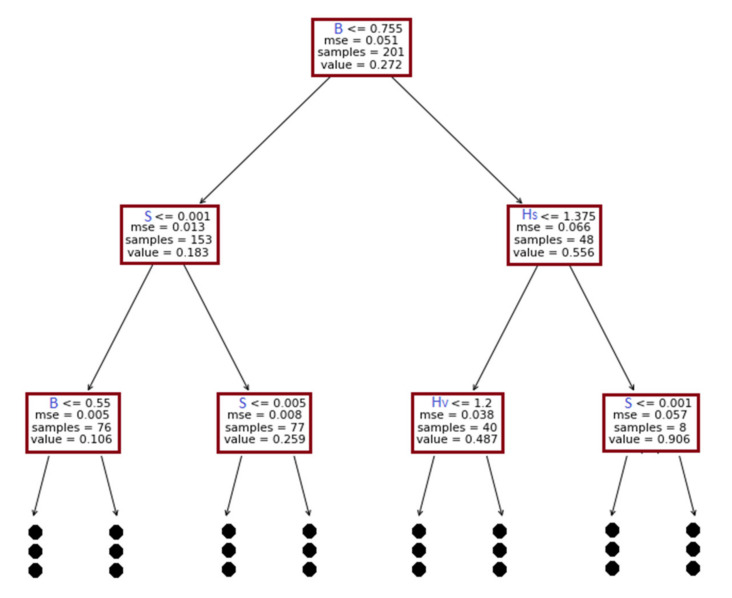
First three layers of the decision tree used in this study.

For tree-based models, the evolved tree structures can be graphically illustrated. However, based on model complexity, the inner-working method is conveniently explainable for models such as simple decision trees. The present study developed separate models for U_B_ and f_s_ predictions. In both phases, the developed decision tree consists of eight layers (tree depth = 8). Regardless of the unique advanced methods used in each tree-based algorithm, the basic decision formation is similar. Therefore, this study presents the first three layers of decision trees formed to predict U_B_ (See Figure 11). 

We suggested a mean squared error (MSE) as the index to perform recursive splits. Therefore, at lower layers, the MSE becomes gradually lower. The tree identifies B as a parameter to start splitting at the root node. Accordingly, a sample that satisfies criteria (B < 0.755) is moved to the left side, whereas the remaining samples are moved to the right side. The term ”value” represents the mean value of the dependent variable that passes through a box. Likewise, the trees continue splitting based on dominant features. For example, in the second level, the tree decides that Hs and S are the dominant features of predictions. At each box, the “IF-THEN” sequence is associated with the left arrow. On one hand, the tree separations are samples with a large variation. However, the tree becomes complex with depth. Therefore, it stresses the requirement for a post-hoc explanation (e.g., SHAP) to explain the inner-working methodology of the ML model. 

### 6.2. Post-Hoc Explanation

**Figure 12:**
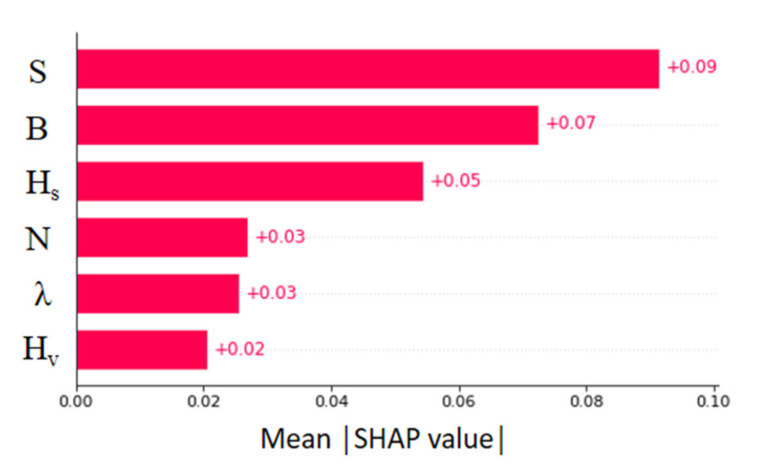
Absolute mean SHAP values of XGBoost model (U_B_ prediction).

**Figure 13:**
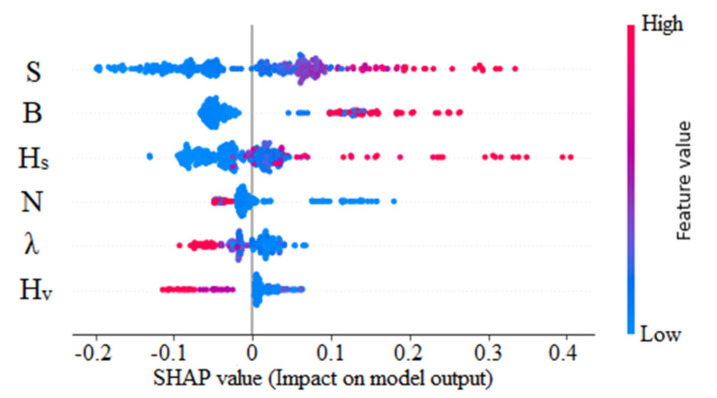
SHAP values of XGBoost model (U_B_ prediction).

Figure 12 shows the average mean absolute SHAP value for the overall U_B_ predictions. Accordingly, the energy slope (S) has the highest impact on U_B_ (+0.09). Next, the effects of channel width (B) and surface height (H_S_) are dominant in the variation of U_B_. Stems (N), vegetation density (λ), and vegetation height (H_V_) have a moderately lower impact on U_B_. The same explanation can be separated into instances to obtain a global explanation as shown in Figure 13. 

Accordingly, the variation is markedly different from the obtained values in Figure 12. For the energy slope, SHAP identifies that lower energy slopes have negative (mostly) and positive impacts on the overall output, whereas higher energy slopes have only a positive impact on the predicted U_B_. The observed influence of channel width (B) is concatenated in the negative region. For example, lower B values result in a negative (low in magnitude) impact on a model’s output. When the channel width increases, a greater positive impact is observed. Interestingly, SHAP notices the dominance of H_S_, whereas an increase in H_S_ may result in a higher positive effect on U_B_. 

However, the effect of N, λ, and H_V_ is in the opposite direction in contrast to previous parameters. For instance, U_B_ decreases when vegetation density increases. A similar effect is observed for the highest of vegetation increases. Thus far, the ML interpretation provides an overview of predictions and their dependence. 

In comparison to the equations proposed to predict U_B_, SHAP conveniently figures out the dominant parameters and their influence in order. Previous models mapped predictions with complex combinations of input features, though an implicit explanation is impossible. However, SHAP provides explanations by mapping the primary parameters where it is convenient to obtain an overall explanation. Further, developing a stepwise regression model requires time and significant expertise and still overlooks the dependence and interactions between parameters. SHAP overcomes such drawbacks and provides the whole resolution within less time. 

**Figure 14:**
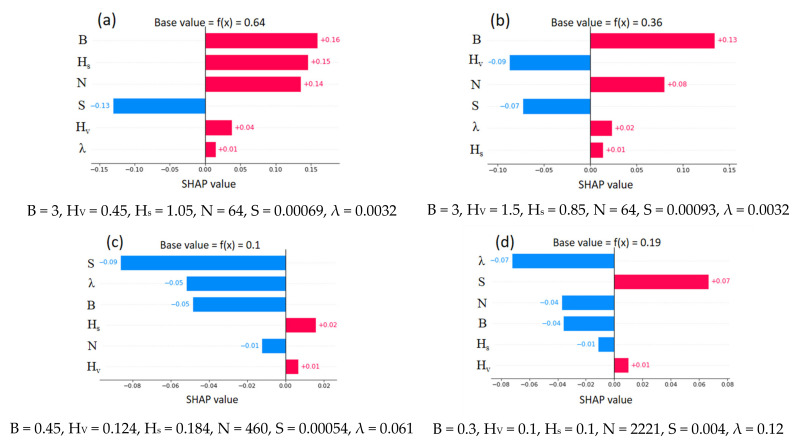
Instance-based SHAP explanations for U_B_ (XGBoost model).

In addition, SHAP provides instance-based explanations with the feature importance value (SHAP value). An instance-based explanation is helpful to distinguish the effect of various parameters in particular instances. Figure 14 shows explanations of four selected instances. The instance-based explanations are notably different from the global explanation. For example, the energy slope, which was the dominant feature of global interpretation, is not a key feature in each instance. From Figure 14a,b, channel width holds major importance. Interestingly, the height of vegetation (H_V_) increases from 0.45 to 1.5, which decreases U_B_ from 0.64 to 0.36. The negative impact of an increase in vegetation height majorly contributes to a change in the *base value* (average value observed during training) of U_B_. The effect of the remaining parameters (Figure 14b) has changed slightly with respect to Figure 14a. From Figure 14c,d, the increase in U_B_ (0.1 to 0.19) depends on several factors. For example, the energy slope changes from S = 0.00054 to S = 0.004, creating a significant positive impact on the output. Simultaneously, an increase in λ from 0.061 to 0.12 creates a slightly pronounced negative effect on U_B_. In both instances, the height of vegetation holds an inferior significance.

**Figure 15:**
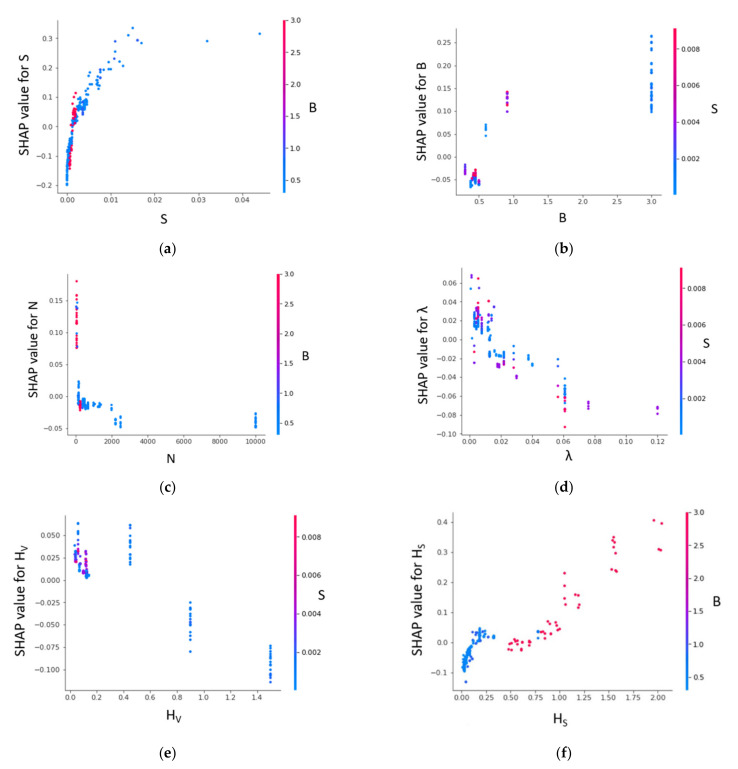
SHAP dependence plot for XGBoost model (U_B_ prediction).

Figure 15 shows a SHAP dependence plot. If a particular feature (first) is selected, it determines the subsequent feature that interacts the most with the selected feature. The y-axis represents the SHAP value of the selected feature. For example, the energy slope (Figure 15a) mostly interacts with channel width (B). However, only the lower features of B interact with higher features of S. The SHAP value drastically increases from 0 < S < 0.01 and becomes stalled. Features N and H_S_ also interact more with feature B. However, the interaction is notably different from that observed in Figure 15a. The SHAP value of N reduces with the feature value. The lower features of N interact more with the higher features of B (Figure 15c). In addition, the lower feature of N obtained SHAP values of mostly less than 0. It is noteworthy that a fair correlation is observed between the SHAP value of H_S_ and feature values of H_S_. H_S_ values in the range of (0.5 to 2) interact with higher features of B. For λ, B, and H_V_, the most interacted variable is the energy slope (S). A weak negative correlation is observed between λ and its SHAP value. Except for λ, frequent interaction is noticed between the higher features of H_V_ and S as well as B and S. 

**Figure 16:**
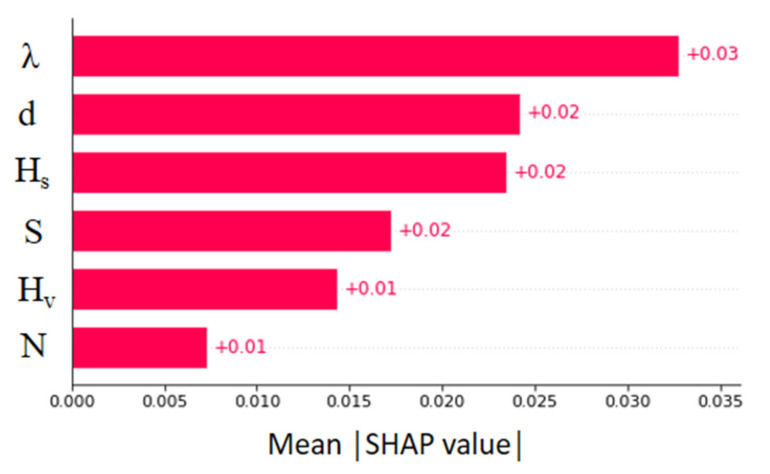
Mean absolute SHAP values of XGBoost model (f_S_ prediction).

Figure 16 shows the absolute mean SHAP value obtained for the f_S_ predictions. The effect of the vegetation density is noticed as dominant. Next, the vegetation diameter and surface layer height have obtained comparable SHAP values. The effect of the vegetation diameter was omitted from the U_B_ prediction model as it held a trivial contribution during the training process. Similarly, the effect of channel width was inferior for f_S_ predictions. The energy slope, which held the most impact for U_B_ predictions, less affects the overall output. The lowest feature contribution was obtained for the number of stems.

**Figure 17:**
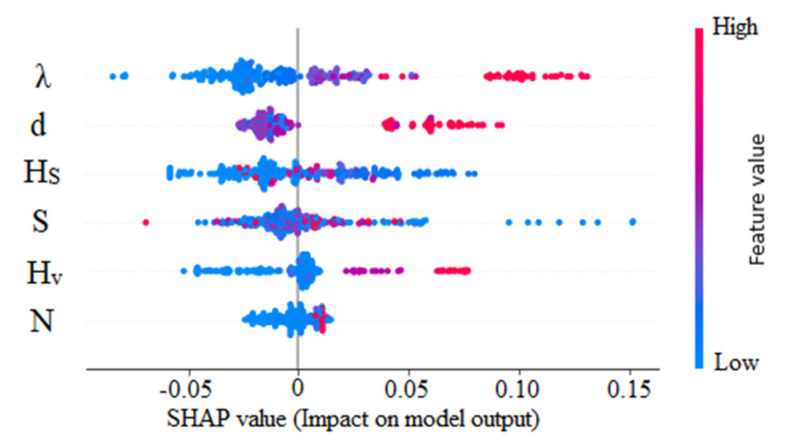
SHAP values of XGBoost model (f_S_ prediction).

All three (d, H_S_, and S) have a mixed impact on model output (See Figure 17). For example, both the lower and higher feature values contribute positive and negative impacts on f_S_. Higher features of vegetation density can contribute up to a 0.15 SHAP value, whereas the lowest feature values achieve a SHAP value of −0.1. Instances exist where lower energy slopes contribute a higher positive effect on the model output compared to vegetation density. On the contrary, the effect of H_V_ and λ is explicitly reversed for f_S_ in contrast to U_B_. A higher vegetation height can increase f_S_.

**Figure 18:**
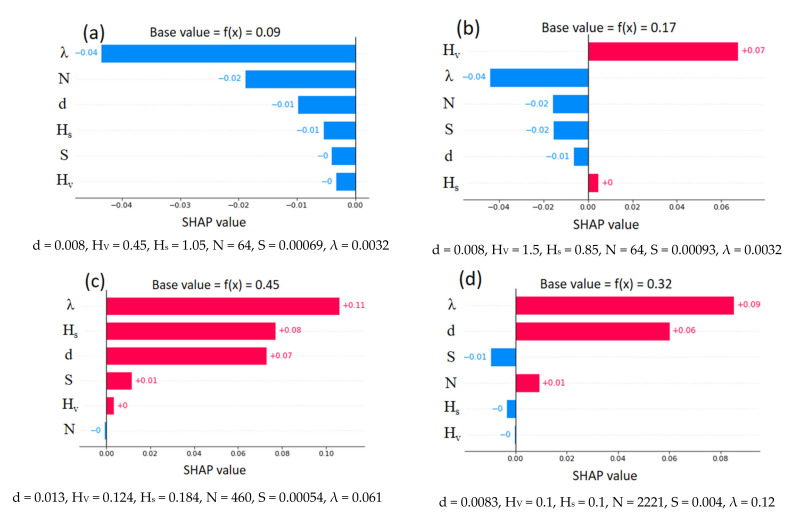
Instance-based SHAP explanation for f_S_ (XGBoost model).

The same four instances that were explained previously were selected for the present explanation (Figure 18). Across three instances, vegetation density shows maximum impact regardless of its direction (negative/positive). From Figure 18a,b, an increase in vegetation height results in a decrease in f_S_. The SHAP value has changed from almost zero to +0.07. This influence corresponds to an increase in f_S_ from 0.09 to 0.17. Likewise, a slight decrease in surface height increases the f_S_ (Figure 18b). When λ is increased from 0.061 to 0.12, the corresponding SHAP value decreased from 0.11 to 0.09. However, an increase in Hs value from 0.1 to 0.184 results in a positive impact on the f_S_. All four instances indicate an increase in the SHAP value (from negative to positive) when the number of stems per unit bed area (N) increases. The effect of the energy slope is almost comparable in magnitude for all four cases.

**Figure 19:**
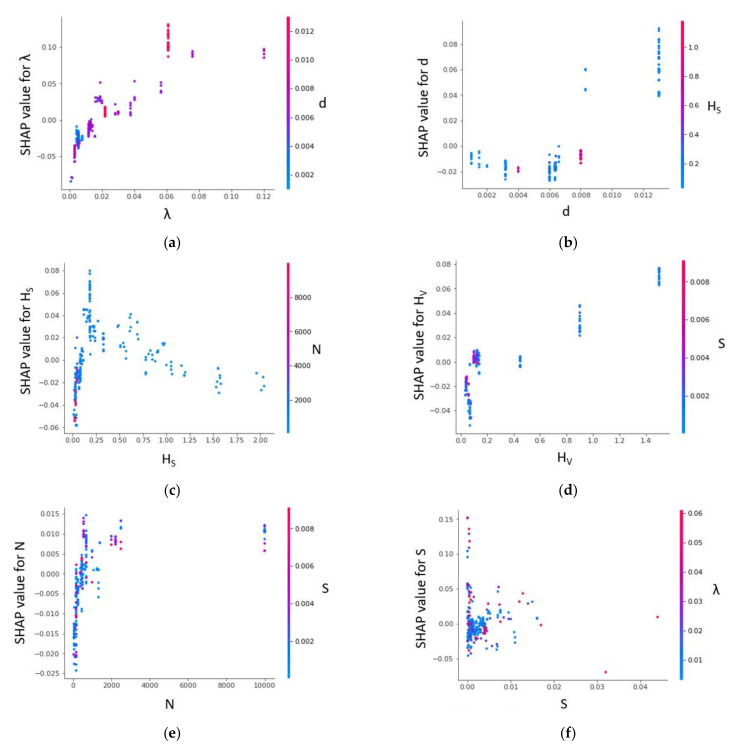
SHAP dependence plot for XGBoost model (f_S_ prediction).

Figure 19 showcases that, for the same data set, two prediction models can consist of different interactions. The vegetation density and energy slope exhibit a moderate correlation with their corresponding SHAP values. However, their interaction with the second feature (d and λ) is a mixed variation. All features of H_S_ mostly interact with the lower features of N (Figure 19c). A similar observation is noticed between d and H_S_ as well as H_V_ and S (Figure 19b,d). Despite the weak linear correlation, a mixed interaction is observed between N and S (Figure 19e).

We highlighted that the SHAP explanations are vital to understanding the complex behavior of flow with vegetation. For example, unique plots (e.g., global explanation, instance-based explanations, the dependence of features, the interaction between features, and feature importance) provide different insights into the prediction model. In contrast, previous regression models fail to elucidate their predictions or the importance of each feature. The confidence in ML-based prediction models increases in the presence of human-comprehensible explanations and causality of predictions. Therefore, we strongly believe that these explanations appeal to the interest of domain experts and improve the end-users’ confidence. In addition, a person with minimum technical knowledge can understand the provided explanation with basic parameters. 

## 7. Conclusions

The following remarks are important findings from this study, which proposed employing XAI and ML to predict the bulk average velocity of open channels with rigid vegetation:

- Ordinary (decision tree) and ensemble tree-based models (extra tree and XGBoost) are accurate in predicting the bulk average velocity (U_B_). However, XGBoost showcased a superior performance, even when compared to existing regression models (R = 0.984). Further, the XGBoost model is accurate in predicting the friction coefficient of the surface layer (f_S_) with an accuracy of R = 0.92. Compared to existing regression models, XGBoost provides consistent predictions under sparse vegetation conditions (λ << 0.1). However, as a result of the complex tree structure, a post-hoc explanation was required to elucidate the XGBoost predictions.
- SHAP revealed the inner-working of the XGBoost model and the underlying reasoning behind respective predictions. Explanations present the contribution of each feature in a model in whole and instances, identifying the dominant parameters. SHAP provides the causality of predictions compared to existing complex regression models without sacrificing either the accuracy or complexity of ML models. Knowledge obtained through SHAP can be used to validate models using experimental data. For example, SHAP explanations adhere to what is generally observed in complex flow with rigid vegetation. Therefore, we believe that it will improve end-users’ and ”domain experts’” trust in implementing ML in hydrology-related studies.

## 8. Limitation and Future Work

This study presented valuable insights on employing explainable artificial intelligence (XAI) with tree-based ML to interpret the rationale behind the bulk average velocity predictions in an open channel with rigid vegetation. However, we highlight the limitations of the present study to conduct future research work in this area:

- The work proposed was focused on open channel flow with rigid vegetation. However, results do not rule out methods to be used with flexible vegetation. A separate study can be carried out using experimental data and explainable ML. It provides a great opportunity to explain the underlying reasoning behind complex applications. Further, the ability of XAI and ML can be explored in hydrology-related applications.
- We suggested tree-based ordinary and ensemble methods as the optimization is more convenient. Further, these models follow a deterministic and human-comprehensible process compared to a neural network. However, several researchers have already used ANN models for hydrology-related studies. Therefore, we suggest examining the performance of advanced ML architectures, such as deep neural networks, generative adversarial networks (GAN), and artificial neural networks (ANN), for the proposed work. These studies can be combined with XAI to obtain the inner workings of the model to improve end-users’ and domain experts’ trust in these advanced ML models.
- It is important to evaluate different explanation models other than SHAP. For example, Moradi and Samweld [69] reported that the explanation process of LIME is markedly different from that of SHAP. The knowledge of different explanation (post-hoc) methods will assist in comparing a set of obtained predictions (feature importance).

## Figures and Tables

**Table 1:** Descriptive statistics of the data set.

	Description	Mean	Maximum	Minimum	Standard Deviation	Kurtosis	Skewness
Q	Measured flow (m^3^/s)	0.58	8.98	0.00	1.45	10.11	3.11
B	Channal width (m)	0.89	3.00	0.38	0.96	1.08	1.72
H	Flow depth (m)	0.52	2.50	0.47	0.69	2.08	1.90
S	Energy slope	0.003	0.044	0.000	0.004	40.88	5.25
λ	Vegetation density (fraction of bed area with stemps)	0.020	0.120	0.020	0.022	5.22	2.16
d	Characteristic diameter of vegetation (m)	0.007	0.013	0.006	0.004	−0.67	0.33
H_V_	Height of vegetation layer (m)	0.24	1.50	0.14	0.36	5.77	2.61
N	Stems per unit bed area (m^−2^)	1210	9995	625	2468	8.4	3.1
H_s_	Height of surface layer (m)	0.28	2.04	0.33	0.40	5.63	2.40
U_B_	Bulk average flow (m/s)	0.28	1.24	0.03	0.22	2.82	1.57

**Table 2:** Comparison of uncertainty indices for validation predictions.

Prediction	Model	R	R^2^	MAE	RMSE	Fractional Bias
U_B_	Decision Tree	0.882	0.78	0.067	0.102	−0.037
	Extra tree	0.944	0.89	0.053	0.071	0.010
	XGBoost	0.984	0.97	0.025	0.040	−0.019
	Shi et al., (2019) [4]	0.970	0.94	0.032	0.053	0.006
	Cheng, (2015) [5]	0.960	0.92	0.040	0.063	−0.023
	Huthoff et al., (2007) [8]	0.972	0.95	0.038	0.060	−0.116
f_S_	Decision Tree	0.761	0.58	0.060	0.096	−0.061
	Extra tree	0.798	0.64	0.055	0.092	−0.057
	XGBoost	0.920	0.85	0.042	0.060	−0.035
	Shi et al., (2019) [4]	0.676	0.46	0.069	0.110	−0.113

**Figure A1:**
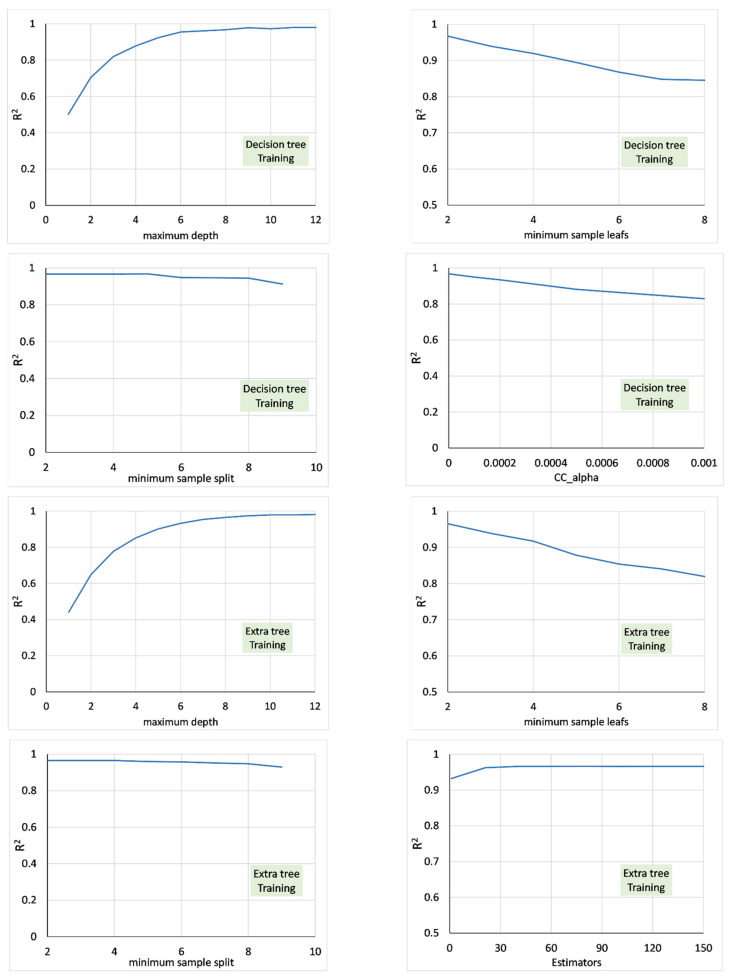

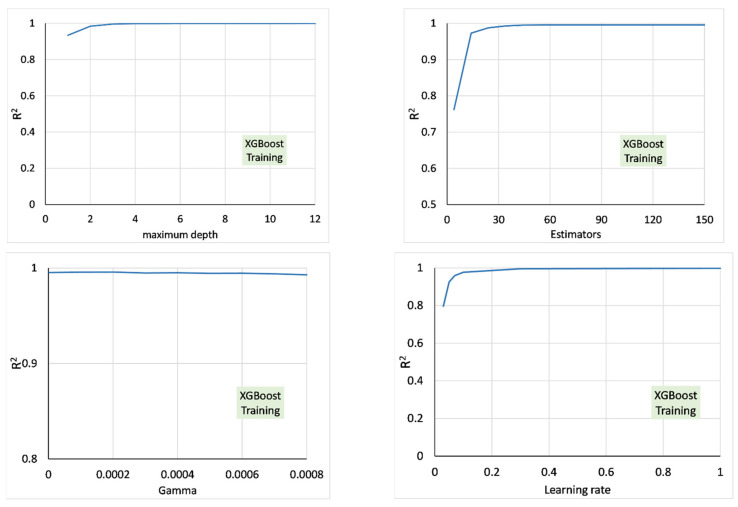
Hyperparameter tuning of tree-based models.

## Data Availability

Data supporting the analysis can be found at https://data.mendeley.com/datasets/kymskr5wjg/1 (accessed on 26 February 2022).

## Appendix A

### Hyperpatameter Tuning

**Table A1:** Optimized/default hyperparameters of tree-based models.

Decision Tree		Extra Tree		XGBoost	
Hyperparameter	Optimized/Assigned Value	Hyperparameter	Optimized/Assigned Value	Hyperparameter	Optimized/Assigned Value
criterion	Mean square error	criterion	Mean square error	Maximum depth	3
splitter	Best	Maximum depth	8	Gamma	0.0002
Maximum depth	8	Minimum samples leaf	2	Learning rate	0.3
Minimum samples leaf	2	Minimum sample split	2	Number of Estimators	50
Minimum sample split	2	Number of Estimators	50	Random state	154
Maximum Features	5	Bootstrap	FALSE	Reg_Alpha	0.0001
Minimum impurity decrease	0	Minimum impurity decrease	0	Base score	0.5
Random state	5464	Random state	5464		
CC alpha	0	Number of jobs	none		

## Appendix B

Performance and associated uncertainty of ML-based prediction should be evaluated with respect to the original sample. For this purpose, we have employed four indices namely, coefficient of correlation (R^2^), Coefficient of determination (R), Mean absolute error (MAE), Root mean square error (RMSE), and Fractional Bias (Equations (A1)–(A5)). Further, these indices will assist in determining the best model in terms of overall performance.

(A1) $$R^{2}=\frac{\sum_{i=1}^{N}{\left(P_{i}-O_{i}\right)}^{2}}{\sum_{i=1}^{N}{\left(P_{i}-{\bar{O}}_{i}\right)}^{2}}$$

(A2) $$R=\frac{N\sum_{i=1}^{N}\left(P_{i}.O_{i}\right)-(\sum_{i=1}^{N}P_{i}.\sum_{i=1}^{N}O_{i})}{\sqrt{\left(N\sum_{i=1}^{N}O_{i}{}^{2}-{\left(\sum_{i=1}^{N}O_{i}\right)}^{2}\right)}.\sqrt{N\sum_{i=1}^{N}P_{i}{}^{2}-{\left(\sum_{i=1}^{N}P_{i}\right)}^{2})}}$$

(A3) $$\mathrm{MAE}=\frac{\sum_{i=1}^{N}\left|O_{i}-P_{i}\right|}{N}$$

(A4) $$\mathrm{RMSE}=\sqrt{\frac{\sum_{i=1}^{N}{\left(O_{i}-P_{i}\right)}^{2}}{N}}$$

(A5) $$\mathrm{Fractional}\;\mathrm{Bias}=\frac{2\left(\bar{P_{i}}-\bar{O_{i}}\right)}{\left(\bar{P_{i}}+\bar{O_{i}}\right)}$$

$P_{i}$ and $O_{i}$ denote prediction and experimental values, respectively. $\bar{O_{i}}$ and $\bar{P_{i}}$ refer to the mean value of the experimental and predicted set.

## References

[1] Huai W.X., Zeng Y.H., Xu Z.G., Yang Z.H. (2009). Three-layer model for vertical velocity distribution in open channel flow with submerged rigid vegetation. Adv. Water Resour..

[2] Nikora N., Nikora V., O’Donoghue T. (2013). Velocity Profiles in Vegetated Open-Channel Flows: Combined Effects of Multiple Mechanisms. J. Hydraul. Eng..

[3] Tang H., Tian Z., Yan J., Yuan S. (2014). Determining drag coefficients and their application in modelling of turbulent flow with submerged vegetation. Adv. Water Resour..

[4] Shi H., Liang X., Huai W., Wang Y. (2019). Predicting the bulk average velocity of open-channel flow with submerged rigid vegetation. J. Hydrol..

[5] Cheng N.-S. (2015). Single-Layer Model for Average Flow Velocity with Submerged Rigid Cylinders. J. Hydraul. Eng..

[6] Tinoco R.O., Goldstein E.B., Coco G. (2015). A data-driven approach to develop physically sound predictors: Application to depth-averaged velocities on flows through submerged arrays of rigid cylinders. Water Resour. Res..

[7] Gualtieri P., De Felice S., Pasquino V., Doria G.P. (2018). Use of conventional flow resistance equations and a model for the Nikuradse roughness in vegetated flows at high submergence. J. Hydrol. Hydromech..

[8] Huthoff F., Augustijn D.C.M., Hulscher S.J.M.H. (2007). Analytical solution of the depth-averaged flow velocity in case of submerged rigid cylindrical vegetation. Water Resour. Res..

[9] Baptist M., Babovic V., Uthurburu J.R., Keijzer M., Uittenbogaard R., Mynett A., Verwey A. (2007). On inducing equations for vegetation resistance. J. Hydraul. Res..

[10] Cheng N.-S. (2011). Representative roughness height of submerged vegetation. Water Resour. Res..

[11] Stone B.M., Shen H.T. (2002). Hydraulic Resistance of Flow in Channels with Cylindrical Roughness. J. Hydraul. Eng..

[12] Yang W., Choi S.-U. (2010). A two-layer approach for depth-limited open-channel flows with submerged vegetation. J. Hydraul. Res..

[13] Gioia G., Bombardelli F.A. (2002). Scaling and Similarity in Rough Channel Flows. Phys. Rev. Lett..

[14] Augustijn D.C.M., Huthoff F., van Velzen E.H. Comparison of vegetation roughness descriptions. Proceedings of the River Flow 2008-Fourth International Conference on Fluvial Hydraulics.

[15] Nepf H.M. (2012). Flow and Transport in Regions with Aquatic Vegetation. Annu. Rev. Fluid Mech..

[16] Pasquino V., Gualtieri P., Doria G.P. (2016). On Evaluating Flow Resistance of Rigid Vegetation Using Classic Hydraulic Roughness at High Submergence Levels: An Experimental Work. Hydrodynamic and Mass Transport at Freshwater Aquatic Interfaces.

[17] Belcher S.E., Jerram N., Hunt J.C.R. (2003). Adjustment of a turbulent boundary layer to a canopy of roughness elements. J. Fluid Mech..

[18] Govindaraju R.S. (2000). Artificial Neural Networks in Hydrology. II: Hydrologic Applications. J. Hydrol. Eng..

[19] Rajaee T., Khani S., Ravansalar M. (2020). Artificial intelligence-based single and hybrid models for prediction of water quality in rivers: A review. Chemom. Intell. Lab. Syst..

[20] Zounemat-Kermani M., Batelaan O., Fadaee M., Hinkelmann R. (2021). Ensemble machine learning paradigms in hydrology: A review. J. Hydrol..

[21] Zounemat-Kermani M., Scholz M. (2013). Computing Air Demand Using the Takagi–Sugeno Model for Dam Outlets. Water.

[22] Shin J., Yoon S., Cha Y. (2017). Prediction of cyanobacteria blooms in the lower Han River (South Korea) using ensemble learning algorithms. Desalin. Water Treat..

[23] Singh G., Panda R.K. (2015). Bootstrap-based artificial neural network analysis for estimation of daily sediment yield from a small agricultural watershed. Int. J. Hydrol. Sci. Technol..

[24] Sun W., Lv Y., Li G., Chen Y. (2020). Modeling River Ice Breakup Dates by k-Nearest Neighbor Ensemble. Water.

[25] Cannon A.J., Whitfield P.H. (2002). Downscaling recent streamflow conditions in British Columbia, Canada using ensemble neural network models. J. Hydrol..

[26] Diks C.G.H., Vrugt J.A. (2010). Comparison of point forecast accuracy of model averaging methods in hydrologic applications. Stoch. Environ. Res. Risk Assess..

[27] Li P.-H., Kwon H.-H., Sun L., Lall U., Kao J.-J. (2009). A modified support vector machine based prediction model on streamflow at the Shihmen Reservoir, Taiwan. Int. J. Climatol..

[28] Tiwari M.K., Chatterjee C. (2010). A new wavelet–bootstrap–ANN hybrid model for daily discharge forecasting. J. Hydroinform..

[29] Erdal H.I., Karakurt O. (2013). Advancing monthly streamflow prediction accuracy of CART models using ensemble learning paradigms. J. Hydrol..

[30] Kim D., Yu H., Lee H., Beighley E., Durand M., Alsdorf D.E., Hwang E. (2019). Ensemble learning regression for estimating river discharges using satellite altimetry data: Central Congo River as a Test-bed. Remote Sens. Environ..

[31] Schick S., Rössler O., Weingartner R. (2018). Monthly streamflow forecasting at varying spatial scales in the Rhine basin. Hydrol. Earth Syst. Sci..

[32] Turco M., Ceglar A., Prodhomme C., Soret A., Toreti A., Francisco J.D.-R. (2017). Summer drought predictability over Europe: Empirical versus dynamical forecasts. Environ. Res. Lett..

[33] Arabameri A., Saha S., Chen W., Roy J., Pradhan B., Bui D.T. (2020). Flash flood susceptibility modelling using functional tree and hybrid ensemble techniques. J. Hydrol..

[34] Li H., Wen G., Yu Z., Zhou T. (2013). Random subspace evidence classifier. Neurocomputing.

[35] Pham B.T., Jaafari A., Van Phong T., Yen H.P.H., Tuyen T.T., Van Luong V., Nguyen H.D., Van Le H., Foong L.K. (2021). Improved flood susceptibility mapping using a best first decision tree integrated with ensemble learning techniques. Geosci. Front..

[36] Shu C., Burn D.H. (2004). Artificial neural network ensembles and their application in pooled flood frequency analysis. Water Resour. Res..

[37] Araghinejad S., Azmi M., Kholghi M. (2011). Application of artificial neural network ensembles in probabilistic hydrological forecasting. J. Hydrol..

[38] Lee S., Kim J.-C., Jung H.-S., Lee M.J., Lee S. (2017). Spatial prediction of flood susceptibility using random-forest and boosted-tree models in Seoul metropolitan city, Korea. Geomat. Nat. Hazards Risk.

[39] Singh K.P., Gupta S., Mohan D. (2014). Evaluating influences of seasonal variations and anthropogenic activities on alluvial groundwater hydrochemistry using ensemble learning approaches. J. Hydrol..

[40] Barzegar R., Fijani E., Moghaddam A.A., Tziritis E. (2017). Forecasting of groundwater level fluctuations using ensemble hybrid multi-wavelet neural network-based models. Sci. Total Environ..

[41] Avand M., Janizadeh S., Tien Bui D., Pham V.H., Ngo P.T.T., Nhu V.-H. (2020). A tree-based intelligence ensemble approach for spatial prediction of potential groundwater. Int. J. Digit. Earth.

[42] Chen W., Zhao X., Tsangaratos P., Shahabi H., Ilia I., Xue W., Wang X., Bin Ahmad B. (2020). Evaluating the usage of tree-based ensemble methods in groundwater spring potential mapping. J. Hydrol..

[43] Belle V., Papantonis I. (2021). Principles and Practice of Explainable Machine Learning. Front. Big Data.

[44] Roscher R., Bohn B., Duarte M.F., Garcke J. (2020). Explainable Machine Learning for Scientific Insights and Discoveries. IEEE Access.

[45] Xu F., Uszkoreit H., Du Y., Fan W., Zhao D., Zhu J. (2019). Explainable AI: A Brief Survey on History, Research Areas, Approaches and Challenges. Natural Language Processing and Chinese Computing.

[46] Hu X., Shi L., Lin G., Lin L. (2021). Comparison of physical-based, data-driven and hybrid modeling approaches for evapotranspiration estimation. J. Hydrol..

[47] Wang S., Peng H., Liang S. (2022). Prediction of estuarine water quality using interpretable machine learning approach. J. Hydrol..

[48] Ahmad M.A., Eckert C., Teredesai A. Interpretable Machine Learning in Healthcare. Proceedings of the 2018 ACM International Conference on Bioinformatics, Computational Biology, and Health Informatics.

[49] Sagi O., Rokach L. (2020). Explainable decision forest: Transforming a decision forest into an interpretable tree. Inf. Fusion.

[50] Lundberg S.M., Lee S.-I. A unified approach to interpreting model predictions. Proceedings of the 31st International Conference on Neural Information Processing Systems.

[51] Liang Y., Li S., Yan C., Li M., Jiang C. (2021). Explaining the black-box model: A survey of local interpretation methods for deep neural networks. Neurocomputing.

[52] Patro B.N., Lunayach M., Patel S., Namboodiri V.P. (2019). U-CAM: Visual Explanation Using Uncertainty Based Class Activation Maps. https://openaccess.thecvf.com/content_ICCV_2019/html/Patro_U-CAM_Visual_Explanation_Using_Uncertainty_Based_Class_Activation_Maps_ICCV_2019_paper.html.

[53] Selvaraju R.R., Cogswell M., Das A., Vedantam R., Parikh D., Batra D. Grad-CAM: Visual Explanations from Deep Networks via Gradient-Based Localization. Proceedings of the 2017 IEEE International Conference on Computer Vision (ICCV).

[54] Zhou B., Khosla A., Lapedriza A., Oliva A., Torralba A. Learning deep features for discriminative localization. Proceedings of the IEEE Conference on Computer Vision and Pattern Recognition.

[55] Ross A., Doshi-Velez F. Improving the adversarial robustness and interpretability of deep neural networks by regularizing their input gradients. Proceedings of the AAAI Conference on Artificial Intelligence.

[56] Zeiler M.D., Fergus R. (2014). Visualizing and understanding convolutional networks. Computer Vision–ECCV 2014, Proceedings of the 13th European Conference, Zurich, Switzerland, 6–12 September 2014.

[57] Binder A., Montavon G., Lapuschkin S., Müller K.R., Samek W. (2016). Layer-wise relevance propagation for neural networks with local renormalization layers. Artificial Neural Networks and Machine Learning–ICANN 2016.

[58] Sundararajan M., Taly A., Yan Q. Axiomatic attribution for deep networks. Proceedings of the 34th International Conference on Machine Learning.

[59] Zhang J., Bargal S.A., Lin Z., Brandt J., Shen X., Sclaroff S. (2018). Top-Down Neural Attention by Excitation Backprop. Int. J. Comput. Vis..

[60] Zhang Q., Wu Y.N., Zhu S.-C. (2018). Interpretable Convolutional Neural Networks. https://openaccess.thecvf.com/content_cvpr_2018/html/Zhang_Interpretable_Convolutional_Neural_CVPR_2018_paper.html.

[61] Ghorbani A., Wexler J., Zou J., Kim B. (2019). Towards Automatic Concept-based Explanations. arXiv.

[62] Zhou B., Sun Y., Bau D., Torralba A. (2018). Interpretable Basis Decomposition for Visual Explanation. Computer Vision–ECCV 2018.

[63] Etmann C., Lunz S., Maass P., Schoenlieb C. On the Connection between Adversarial Robustness and Saliency Map Interpretability. Proceedings of the 36th International Conference on Machine Learning.

[64] Tao G., Ma S., Liu Y., Zhang X. (2018). Attacks Meet Interpretability: Attribute-steered Detection of Adversarial Samples. arXiv.

[65] Aydin Y., Dizdaroğlu B. (2020). Blotch Detection in Archive Films Based on Visual Saliency Map. Complexity.

[66] Fong R.C., Vedaldi A. Interpretable Explanations of Black Boxes by Meaningful Perturbation. Proceedings of the 2017 IEEE International Conference on Computer Vision (ICCV).

[67] Ribeiro M.T., Singh S., Guestrin C. “Why Should I Trust You?” Explaining the Predictions of Any Classifier. Proceedings of the 22nd ACM SIGKDD International Conference on Knowledge Discovery and Data Mining.

[68] Petsiuk V., Das A., Saenko K. (2018). RISE: Randomized Input Sampling for Explanation of Black-box Models. arXiv.

[69] Moradi M., Samwald M. (2021). Post-hoc explanation of black-box classifiers using confident itemsets. Expert Syst. Appl..

[70] Baptist M.J. (2005). Modelling Floodplain Biogeomorphology. https://repository.tudelft.nl/islandora/object/uuid%3Ab2739720-e2f6-40e2-b55f-1560f434cbee.

[71] Dunn C., Lopez F., Garcia M.H. (1996). Mean Flow and Turbulence in a Laboratory Channel with Simulated Vegatation (HES 51). https://www.ideals.illinois.edu/handle/2142/12229.

[72] Liu D., Diplas P., Fairbanks J.D., Hodges C.C. (2008). An experimental study of flow through rigid vegetation. J. Geophys. Res. Earth Surf..

[73] Meijer D.G., van Velzen E.H. Prototype-Scale Flume Experiments on Hydraulic Roughness of Submerged Vegetation. Proceedings of the 28th IAHR Congress.

[74] Murphy E., Ghisalberti M., Nepf H. (2007). Model and laboratory study of dispersion in flows with submerged vegetation. Water Resour. Res..

[75] Poggi D., Porporato A., Ridolfi L., Albertson J.D., Katul G. (2004). The Effect of Vegetation Density on Canopy Sub-Layer Turbulence. Bound. Layer Meteorol..

[76] Shimizu Y., Tsujimoto T., Nakagawa H., Kitamura T. (1991). Experimental study on flow over rigid vegetation simulated by cylinders with equi-spacing. Doboku Gakkai Ronbunshu.

[77] Yang W. (2008). Experimental Study of Turbulent Open-channel Flows with Submerged Vegetation. Ph.D. Thesis.

[78] Xu M., Watanachaturaporn P., Varshney P.K., Arora M.K. (2005). Decision tree regression for soft classification of remote sensing data. Remote Sens. Environ..

[79] Breiman L., Friedman J.H., Olshen R.A., Stone C.J. (1984). Classification and Regression Trees.

[80] Ahmad M.W., Reynolds J., Rezgui Y. (2018). Predictive modelling for solar thermal energy systems: A comparison of support vector regression, random forest, extra trees and regression trees. J. Clean. Prod..

[81] Rodriguez-Galiano V., Sanchez-Castillo M., Chica-Olmo M., Chica-Rivas M.J.O.G.R. (2015). Machine learning predictive models for mineral prospectivity: An evaluation of neural networks, random forest, regression trees and support vector machines. Ore Geol. Rev..

[82] Maree R., Geurts P., Piater J., Wehenkel L. A Generic Approach for Image Classification Based on Decision Tree Ensembles and Local Sub-Windows. Proceedings of the 6th Asian Conference on Computer Vision.

[83] Geurts P., Ernst D., Wehenkel L. (2006). Extremely randomized trees. Mach. Learn..

[84] Xu C., Liu X., Wang H., Li Y., Jia W., Qian W., Quan Q., Zhang H., Xue F. (2021). A study of predicting irradiation-induced transition temperature shift for RPV steels with XGBoost modeling. Nucl. Eng. Technol..

[85] Chen T., Guestrin C. XGBoost: A Scalable Tree Boosting System. Proceedings of the 22nd ACM SIGKDD International Conference on Knowledge Discovery and Data Mining.

